# TRUST IN GOD AND THE CHURCH, NOT NEIGHBORS: EXPLORING SOCIAL COHESION AMONG AGING HIV+ INDIVIDUALS

**DOI:** 10.1093/geroni/igac059.3023

**Published:** 2022-12-20

**Authors:** Thi Vu, Katherine Dunham, Anna Lin-Schweitzer, Meron Girma, Megan Lee, Yusuf Ransome

**Affiliations:** Yale University, New Haven, Connecticut, United States; Yale School of Public Health, New Haven, Connecticut, United States; Yale School of Public Health, New Haven, Connecticut, United States; Yale University School of Public Health, New Haven, Connecticut, United States; Yale School of Medicine, New Haven, Connecticut, United States; Yale School of Public Health, New Haven, Connecticut, United States

## Abstract

Rates of new HIV diagnoses among people 55+ years old remained unchanged between 2015–2019, suggesting potential HIV prevention challenges in the aging population due to co-morbidities, social isolation, and age-related stigma. Socio-cultural factors play a strong role in influencing HIV outcomes. Social cohesion — including appraisals of trust in neighbors and levels of belongingness to a community — has been shown to be an important channel for HIV prevention in the general population. However, there is scant evidence about how social cohesion relates to HIV prevention and care engagement among older adults. We employed a convergent, parallel mixed-methods study to investigate this topic among a sample (n =17) of adults aged 50+ living with HIV in New Haven, CT. We conducted semi-structured interviews which were analyzed using thematic analysis in NVivo.v12. We also collected quantitative data in RedCap and calculated descriptive statistics in STATA.v16. Participants were on average 57 years of age (SD=4); 53% female, and 69% Black/African American. Participants’ trust in their neighborhoods was low [mean=2.61 (range 1 - 5, where 5 indicates high trust)]. In interviews, trust did not significantly influence one’s HIV status disclosure or care management. Instead, participants often identified their faith in God or relationship with local churches as significant sources of social and informational support they often rely on for their HIV-related needs. Our results show that investigating the impact of religious belonging on outcomes may be a fruitful path of research to improve HIV outcomes among the aging population.

